# Media trust during the COVID-19 pandemic in Ukraine

**DOI:** 10.1192/j.eurpsy.2022.959

**Published:** 2022-09-01

**Authors:** S. Tukaiev, T. Vasheka, V. Rizun, A. Enzminger, Y. Havrylets, B. Palamar, O. Dolgova, O. Pravda, M. Makarchuk

**Affiliations:** 1 National Taras Shevchenko University of Kyiv, Institute Of Biology And Medicine, Kyiv, Ukraine; 2 National Aviation University, Faculty Of Linguistics And Social Communications, Kyiv, Ukraine; 3 National Taras Shevchenko University of Kyiv, Institute Of Journalism, Kyiv, Ukraine; 4 Vienna University of Economics and Business, Nstitute For Communication Management And Media, Vienna, Austria; 5 Bogomolets National Medical University, Department Of Social Medicine And Public Health, Kyiv, Ukraine

**Keywords:** media trust, COVID19 pandemic, youth, psychological portrait

## Abstract

**Introduction:**

The worldwide pandemic exacerbated the new role of the media.If previously the discussion was on whether new or traditional media had primacy in popularity and exposure, nowadays the question is whether communicating health issues through social and traditional media leads to understanding their content better and to more trust in both types of media.

**Objectives:**

We set the following objectives for this study:(1) to examine trust in the traditional and new media among university students,(2) according to the level of media trust to compose a psychological portrait,establish the most prevalent coping strategies,and emotional reactions to the pandemic.

**Methods:**

213 university students (55.9% women,Mage=19 years) were tested from December 2020-March 2021.We examined the attitude towards information on coronavirus presented in the media and to investigate the level of severity of neurotic states,the level of psychological stress,and basic coping strategies used by respondents.

**Results:**

showed that although students generally prefer to use Internet news, trust in traditional media increased during the pandemic. We examined a general psychological portrait of young people derived from trust in the media. In the group of students who trust media information, we found indifference (39% of respondents) and helplessness (24.4%). In the group convinced that the media are hiding the actual state of affairs, anger prevailed (32.4%). The third group, confident that the media exaggerate everything, experienced indifference and anger (38.5% and 32.7%, respectively).

**Conclusions:**

We may conclude that desire to learn more accurate and unbiased information firsthand indicates students’ attitude towards traditional media as more reliable sources of information.

**Disclosure:**

No significant relationships.

